# Photocatalytic Performance of a Novel MOF/BiFeO_3_ Composite

**DOI:** 10.3390/ma10101161

**Published:** 2017-10-10

**Authors:** Yunhui Si, Yayun Li, Jizhao Zou, Xinbo Xiong, Xierong Zeng, Ji Zhou

**Affiliations:** 1 Shenzhen Key Laboratory of Special Functional Materials & Shenzhen Engineering Laboratory for Advance Technology of Ceramics, College of Materials Science and Engineering, Shenzhen University, Shenzhen 518060, China; 2161120218@email.szu.edu.cn (Y.S.); zoujizhao@szu.edu.cn (J.Z.); xxbszdx@szu.edu.cn (X.X.); zengxier@szu.edu.cn (X.Z.); 2 State Key Laboratory of New Ceramics and Fine Processing, School of Materials Science and Engineering, Tsinghua University, Beijing 100084, China; zhouji@tsinghua.edu.cn

**Keywords:** MOF, BiFeO_3_, photocatalysts, morphology

## Abstract

In this study, MOF/BiFeO_3_ composite (MOF, metal-organic framework) has been synthesized successfully through a one-pot hydrothermal method. The MOF/BiFeO_3_ composite samples, pure MOF samples and BiFeO_3_ samples were characterized by X-ray diffraction (XRD), scanning electron microscopy (SEM), energy dispersive spectroscopy (EDS), and by UV–vis spectrophotometry. The results and analysis reveal that MOF/BiFeO_3_ composite has better photocatalytic behavior for methylene blue (MB) compared to pure MOF and pure BiFeO_3_. The enhancement of photocatalytic performance should be due to the introduction of MOF change the surface morphology of BiFeO_3,_ which will increase the contact area with MB. This composing strategy of MOF/BiFeO_3_ composite may bring new insight into the designing of highly efficient photocatalysts.

## 1. Introduction

In the past few decades, much attention has been paid to semi-conductor photocatalysts for their potential utilization of solar energy to solve the increasing environmental and energy crisis [1]. As a first generation photocatalyst, TiO_2_ has attracted the attention of many scientists. In the 1970s, the photocatalytic properties of TiO_2_ were investigated [2]. Due to its high photocatalytic performance, non-toxicity, low cost, and ease of preparation, TiO_2_ has been widely used in water pollution control and hydrogen production by decomposition of water. However, TiO_2_ based photocatalysts are limited by the UV band due to their wide band gap defects [3], and can hardly utilize visible light. While ultraviolet light accounts for less than 5% of sunlight, the utilization efficiency of solar light limits the practical application of TiO_2_ as a photocatalyst. With a narrow band gap of ~2.2 ev and excellent chemical stability, BiFeO_3_ has good response in the visible light range and higher photocatalytic efficiency compared to TiO_2_ based photocatalyst [4]. However, the high recombination rate of photogenerated electrons and holes, poor adsorption, and short lifetime of carriers result in lower photocatalytic activity of pure BiFeO_3_. At present, measures to improve the photocatalytic performance of BiFeO_3_ mainly include adjusting the size and morphology by changing the process [5], ion doping of rare earth elements [6], and surface deposition of noble metals [7]. To some extent, doping of rare earth ions can reduce the band gap of BiFeO_3_ and increase photocatalytic activity [8,9]. However, the complete catalytic activity considerably depends upon surface adsorption over the surface of the photocatalyst [10,11,12]. A great deal of research on the modification of BiFeO_3_ does not improve its adsorption performance significantly. In general, porous materials with different pore sizes have a strong adsorption capacity, which has aroused our interest. Up until now, compositing BiFeO_3_ with porous materials has scarcely been reported.

Metal-organic framework materials (MOFs) have the advantages of ordered pore structure, large specific surface area, and adjustable pore shape and size, their adsorption capacity is much higher than that of conventional adsorbents (such as molecular sieves and activated carbon) [13,14]. ZIF-8, 2-Methylimidazole zinc salt, one of the most widely studied zeolitic imidazolate framework materials, not only has the advantages of the above MOFs, but also has better hydrothermal stability [15], which gives it potential to couple with BiFeO_3_. Benefitting from the diversity of the MOF species, this strategy of MOF/BiFeO_3_ composite would provide new ideas for the designing of highly efficient photocatalysts.

In this work, MOF and BiFeO_3_ composite photocatalyst was successfully synthesized by a simple and low cost hydrothermal process. Photodegradation experiments showed that the composite photocatalyst can effectively improve the photocatalytic ability of degrading organic dye compared with the pure BiFeO_3_ and pure MOF.

## 2. Experimental Section

### 2.1. Preparation of MOF

For synthesis of ZIF-8, 0.744 g (2.50 mmol) Zn(NO_3_)_2_·6H_2_O was dissolved in 100 mL methanol, 0.410 g (5 mmoL) 2-Methylimidazole was dissolved in 160 mL methanol respectively, stirring until fully dissolved. Then the two solutions were mixed by magnetic stirring, the molar ratio of the various components in the mixed solution was maintained at: Zn^2+^:2-Methylimidazole = 1:2. The whole operation was carried out at room temperature without any heating. The solution was stirred with a constant speed at 800 r/min for 1 h, changed from transparent to ivory-white completely. The synthesized powder was washed by methanol and collected by centrifuging, then dried for 12 h in the fume hood.

### 2.2. Preparation of MOF/BiFeO_3_ Composite

The preparation of BiFeO_3_ powder by hydrothermal method has the advantages of controllable morphology, high purity, and small particle size distribution [16]. For synthesis of MOF/BiFeO_3_ composite, Fe(NO_3_)_3_·9H_2_O and Bi(NO_3_)_3_·5H_2_O were added into deionized water with a molar ratio of 1:1. Then it was mixed with 1 mL nitric acid (68 wt %) and 2 g Polyethylene glycol (PEG-2000) by constant stirring at room temperature. 40 mL of KOH (12 M) solution used as a mineralizer was added dropwise into the mixed solution as slowly as possible. When the pH value of the solution was neutral, 0.3 g of prepared ZIF-8 powder was added. Subsequently, the mixture was transferred into a 50 mL Teflon lined stainless vessel, and heated at 180 °C for 24 h. After nature cooling, the obtained sample was centrifuged and thoroughly washed with deionized water. Finally, the MOF/BiFeO_3_ composite was dried at 80 °C in a vacuum oven for 14 h.

### 2.3. Characterization of Phase and Microstructure

The phase compositions were characterized by X-ray powder diffraction (D8Advance, Karlsruhe, Germany) with a graphite monochromator and Cu Kα (λ = 0.15418 nm) radiation operating at 40 kV and 200 mA. The microstructural morphologies of the MOF/BiFeO_3_ composite were observed via scanning electron microscopy (Hitachi SU70, Tokyo, Japan).

### 2.4. Measurements of Photocatalytic Performance

The photocatalytic performance of pure BiFeO_3_, pure MOF, and MOF/BiFeO_3_ photocatalysts were evaluated by the degradation of Methylene blue (MB) with a concentration of 20 mg/L in aqueous solution under visible light irradiation (Xe lamp, 300 W; visible cut off filter >420 nm). Methylene blue is a cationic dye with a methyl nitride group [(CH_3_)_2_N^+^]. In order to prevent any thermal catalytic effect, the reaction temperature was kept at 20 °C by circulation water during the whole process. For intuitive contrast, 30 mg of samples were applied to photocatalytic experiments. The samples and 80 mL MB solution (20 mg/L) were placed in a reactor, and the suspension was placed in the dark and magnetically stirred for 30 min to reach the adsorption/desorption equilibrium before light irradiation. The degradation of MB was examined using a spectrophotometer (Shimadzu UV-2450, Kyoto, Japan) by centrifuging the retrieved samples and measuring the intensity of the absorption peak of MB (663 nm) relative to its initial value (C/C_0_) at intervals of 20 min.

## 3. Results and Discussion

### 3.1. X-ray Diffraction Analysis

**Figure 1:**
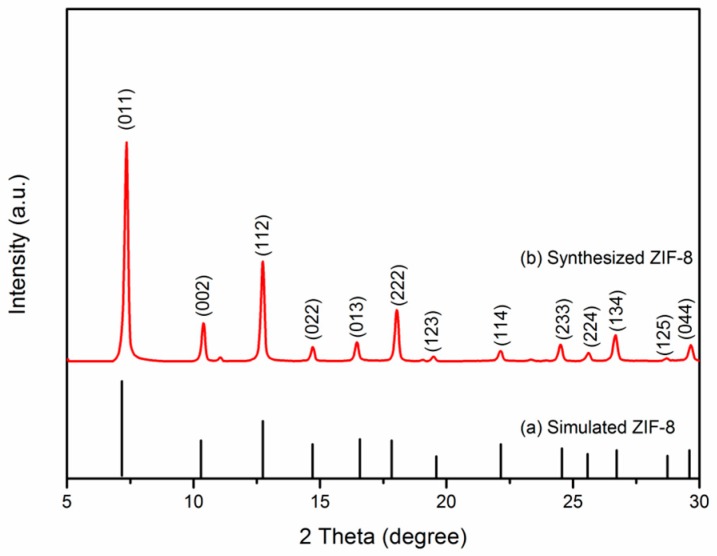
XRD patterns of (**a**) simulated MOF (ZIF-8) and (**b**) synthesized pure MOF (ZIF-8).

Figure 1 shows the X-ray diffraction (XRD) patterns of (a) simulated MOF (ZIF-8) and (b) synthesized MOF (ZIF-8). The diffraction peaks of synthesized MOF sample are in indexed as cubic structure with an I-43 m(217) space group (CCDC number: 602542). The positions and relative intensity of all diffraction peaks match well with simulated MOF. No other impurity peaks are detected, indicating the highly crystalline structure of pure MOF.

**Figure 2:**
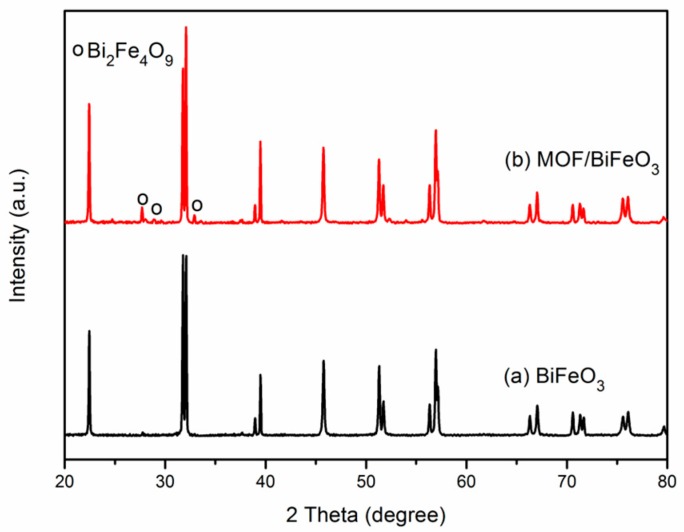
XRD patterns of (**a**) pure BiFeO_3_ and (**b**) MOF/BiFeO_3_.

Figure 2 shows the XRD patterns of MOF/BiFeO_3_ composite samples. The diffraction peaks of MOF/BiFeO_3_ composite in Figure 2b are identified as a perovskite based rhombohedral structure with an R3c space group (JCPDS No. 86-1518) along with the existence of minority phase such as Bi_2_Fe_4_O_9_. It is interesting to note that the composite with MOF does not change the phase structure of BiFeO_3_, and the composite still exhibits almost the same purity and crystallinity as the pure BiFeO_3_ shown in Figure 2a.

### 3.2. Morphology and Microstructure Analysis

**Figure 3:**
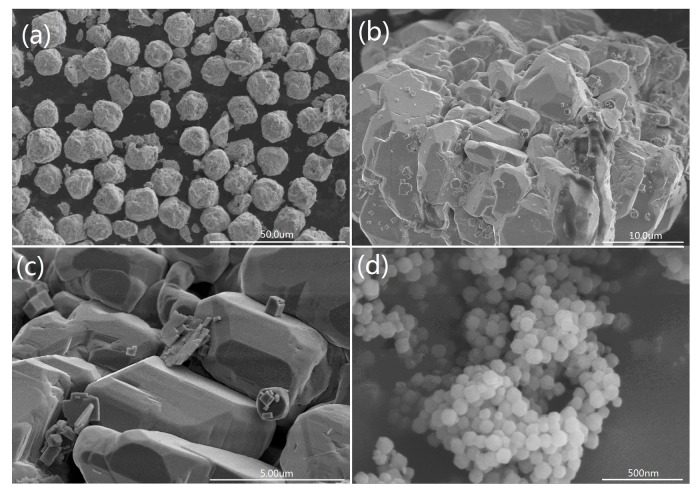
SEM images of (**a**) pure BiFeO_3_; (**b**,**c**) the magnification of pure BiFeO_3_; and (**d**) pure MOF.

**Figure 4:**
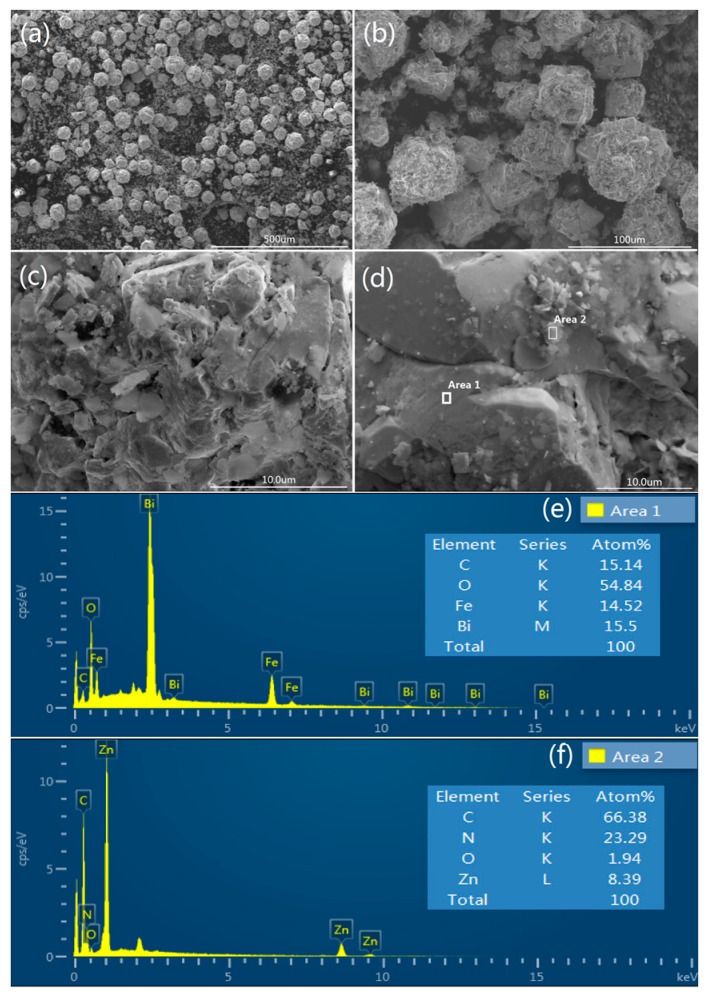
SEM images of (**a**) MOF/BiFeO_3_ composite; (**b**–**d**) the magnification of MOF/BiFeO_3_ composite. EDS spectrum of MOF/BiFeO_3_ composite, (**e**) Area 1, (**f**) Area 2.

As seen in Figure 3a, spherical BiFeO_3_ particles are prepared by a hydrothermal process. Furthermore, no other morphologies can be detected, indicating a high uniformity of the product with the spherical morphology. The magnification of pure BiFeO_3_ shown in Figure 3b,c, spherical BiFeO_3_ are formed by the aggregation of many microcubes, which is consistent with a previous report [17]. It is clear that the surface morphology of BiFeO_3_ particle is very smooth in Figure 3c, which may be one of the reasons for the poor adsorption ability of the pure BiFeO_3_. Figure 3d shows the SEM images of prepared MOF (ZIF-8), pure MOF particles exhibit regular spherical morphology with an average diameter of 60~80 nm. SEM images of MOF/BiFeO_3_ composite in Figure 4a–d show lamellar MOF well disperse on the surface of BiFeO_3_ particle, the surface morphology of MOF/BiFeO_3_ composite changes obviously compared with the pure BiFeO_3_. The particle surface is coarse and porous, which allows the organic molecules to adsorb on the catalyst surface more easily and provide more reactive sites. These will contribute to the increase of the reactive sites, as well as the enhanced separation efficiency of the photogenerated electron–hole pairs.

Energy dispersive spectroscopy (EDS) analysis was carried out to identify the components of MOF/BiFeO_3_ composite. Figure 4e shows the diffraction peaks of Bi, Fe, O, and C elements corresponding to MOF/BiFeO_3_ composite observed in area 1 marked in Figure 4d. From the insert table in Figure 4e, it can be seen that the experimental atomic ratio of Bi, Fe, and O elements is close to the theoretical one of pure BiFeO_3_. A small amount of C element was detected and should be the result of a small amount of MOF adhering to the surface of the BiFeO_3_. Figure 4f shows the element contents of Area 2 marked in Figure 4d. The ratio of the elements in the insert table is close to the theoretical ZIF-8 and confirms that the lamellar MOF was successfully dispersed on the surface of BiFeO_3_ particle.

### 3.3. Enhanced Photocatalytic Performance

**Figure 5:**
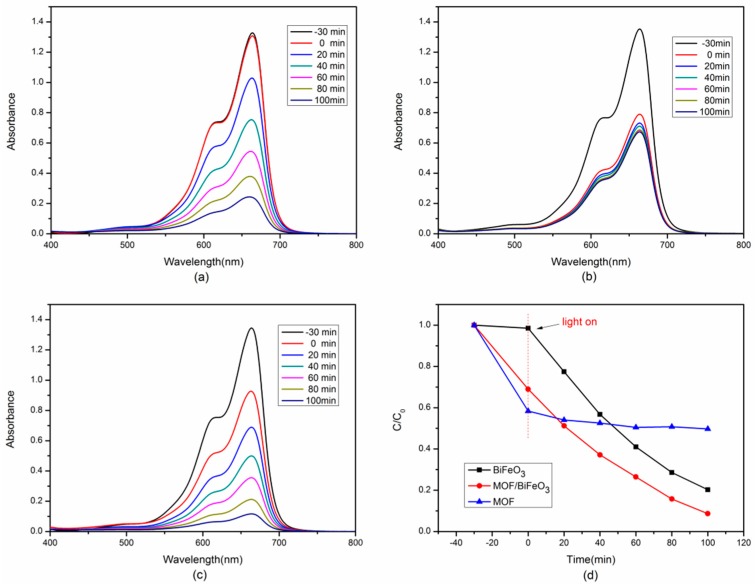
Time dependent UV–vis spectrum changes of MB catalyzed by (**a**) pure BiFeO_3_; (**b**) pure MOF; and (**c**) MOF/BiFeO_3_ composite; (**d**) The photocatalytic degradation efficiencies of all samples to degrade MB.

The photocatalytic activity of pure BiFeO_3_, pure MOF, and MOF/BiFeO_3_ composite photocatalysts under visible light irradiation were defined by measuring the photodegradation of MB aqueous solution. Where C_0_ and C are the initial and final concentration of MB, respectively. After 100 min of visible light irradiation, the maximum percentage of dye decomposition increased from 78% for pure BiFeO_3_ to 93% for MOF/BiFeO_3_ composite photocatalyst, as shown in Figure 5d. As a contrast, when pure MOF powders of the same amount were added to the photocatalytic experiments in Figure 5b, they absorbed about 48% of the dye in the dark environment for 30 min, and hardly underwent photocatalytic reactions under light irradiation. Degradation experiments showed that MOF has no photocatalytic capacity, despite excellent adsorption properties. After 40 min, dye adsorption reached the maximum, 50% of the MB dye was adsorbed. As shown in Figure 5a,c, compared with pure BiFeO_3_, the enhancement of MB photodegradation efficiency for MOF/BiFeO_3_ composite photocatalyst was mainly thanks to the promotion of adsorption capability. Approximately 32% MB was absorbed by composite photocatalyst in the first 30 min. However, for pure BiFeO_3_, only 1.5% was absorbed. The results of degradation experiments confirmed that the change of the surface morphology of the MOF/BiFeO_3_ composite photocatalyst can effectively enhance the photocatalytic degradation ability of MB molecules.

## 4. Photocatalytic Reaction Mechanism of MB over MOF/BiFeO_3_ Composite

**Figure 6:**
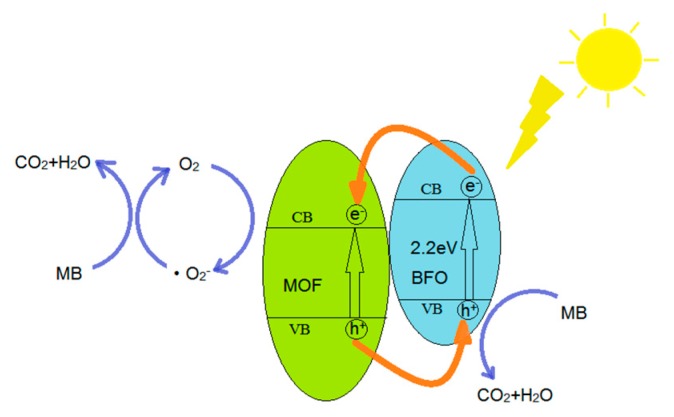
Schematic diagram for photocatalytic degradation of MB.

Under the irradiation of visible light, MOF/BiFeO_3_ composite photocatalyst can effectively absorb photons thanks to its narrow band gap. The photocatalytic process included a series of photochemical reactions through the first step of electrons (e^−^) and holes (h^+^) generation (Figure 6). The photogenerated holes in the valence band (VB) combine with absorbed H_2_O molecules to form strong oxidized hydroxyl radical (OH). In the presence of dissolved O_2_, the electrons in the conduction band (CB) can react with O_2_ to form superoxide radical (O_2_^−^) and hydrogen peroxide (H_2_O_2_). The OH radical has been deliberated to be the key active species accountable for the BiFeO_3_ photocatalytic process. The holes can also directly react with organic pollutants adsorbed on MOF/BiFeO_3_ composite and oxidize it to CO_2_ and H_2_O. The proposed mechanism (1–8) is as follows:

1. Absorption of efficient photons by MOF/BiFeO_3_ composite photocatalyst
  (1) $$(\mathrm{MOF}/\mathrm{BiFe}O_{3})+h\nu \to e_{\mathrm{CB}}^{-}+h_{\mathrm{VB}}^{+}$$
2. Holes (h^+^) in valence band combine with absorbed H_2_O molecules which produces hydroxyl radical (·OH)
  (2) $$h^{+}+H_{2}O\to •\mathrm{OH}+H^{+}$$
3. Oxygen ionosorption
  (3) $$O_{2}+e^{-}\to •O_{2}^{-}$$
4. Neutralization of by $•O_{2}^{-}$ protons
  (4) $$•O_{2}^{-}+H^{+}\to •\mathrm{OOH}$$
5. Transient hydrogen peroxide formation and dismutation of oxygen
  (5) $$2•\mathrm{OOH}\to O_{2}+H_{2}O_{2}$$
6. Decomposition of hydrogen peroxide
  (6) $$H_{2}O_{2}+e^{-}\to •\mathrm{OH}+OH^{-}$$
7. Hydroxyl radical is further generated
  (7) $$H_{2}O_{2}+•O_{2}^{-}\to •\mathrm{OH}+OH^{-}+O_{2}$$
8. Oxidation of the MB molecules via successive attacks by **·**OH and direct oxidation by reaction with holes
  (8) $$\mathrm{MB}+•\mathrm{OH},\;h^{+}\to CO_{2}+H_{2}O+\cdot \cdot \cdot \cdot \cdot \cdot$$

## 5. Conclusions

In this work, a novel MFO/BiFeO_3_ composite photocatalyst with high photocatalytic efficiency has been successfully synthesized through a hydrothermal method. Characterization of the composite photocatalyst confirmed that the MOF with strong adsorptive property was successfully immobilized on the BiFeO_3_ structure. The surface morphology of MOF/BiFeO_3_ composite changed obviously compared with the pure BiFeO_3_, a large surface area increased the number of active sites to promote the separation of e^−^ and h^+^ pairs and improved the light absorption ability owing to multiple scattering effect. According to the degradation of MB, the introduction of MOF can enhance the adsorption capacity of BiFeO_3_ effectively, thereby enhancing the degradation of MB under visible light irradiation. This work proposed a new idea for efficient photocatalysts that can be used for purification of industrial waste.
